# Ovarian Serous Cystadenoma of the Epididymis: A Case Report

**DOI:** 10.7759/cureus.46886

**Published:** 2023-10-12

**Authors:** Daniel P Pierce, Bo Chen, Laura Taylor

**Affiliations:** 1 Urology, University of South Florida (USF) Health, Tampa, USA; 2 Department of Pathology and Cell Biology, University of South Florida (USF) Morsani College of Medicine, Tampa, USA

**Keywords:** ovarian serous cystadenoma, serous borderline tumor, paratesticular mass, epididymis, extratesticular

## Abstract

Paratesticular cystadenomas remain a very rare entity, typically presenting as a painless mass, often indistinguishable from the testicle. As such, the predominant management seems to be complete excision via various approaches, which often proves curative. Given its rarity, post-operative surveillance has not been standardized; most patients and providers elect a more conservative surveillance approach. Based on the available literature, this seems appropriate, given the lack of morbidity or recurrence associated with these types of tumors.

## Introduction

Cystic structures of the testis and epididymis are often benign and vary in etiology. The most common benign primary tumor of the epididymis is the adenomatoid tumor, followed by papillary cystadenoma [1]. The literature review yields less than 10 case reports of testicular or para-testicular ovarian or pure serous type tumors and even fewer of epididymal origin. We present a case of a 65-year-old male who presented with a painless swelling of the right epididymis.

## Case presentation

**Figure 1:**
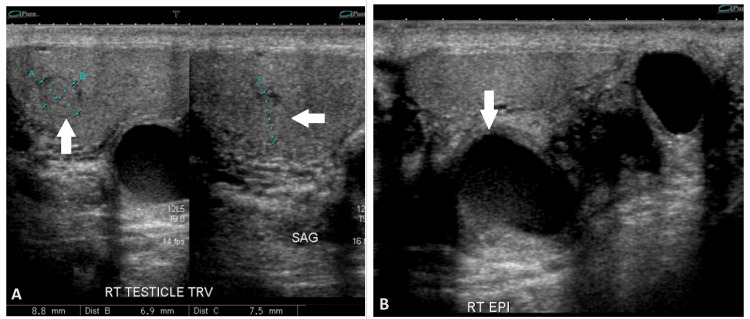
Ultrasound images of the right testicle A: Coronal and sagittal ultrasound view of the right testicle demonstrating a 9mm hypoechoic right testicular lesion as measured above. B: 2cm right-sided spermatocele within the epididymis.

A 65-year-old male with a history of medically managed atrial fibrillation incidentally discovered a firm right para-testicular mass that resulted in intermittent right groin discomfort for six months. Physical examination was notable for normal and descended testicles bilaterally, a right palpable para-testicular mass that was mildly tender on palpation, a cyst of the left epididymal head, and no other obvious testicular or scrotal lesions. The patient ultimately underwent a scrotal sonogram demonstrating what was originally suggested to be a 2cm right-sided spermatocele and a 1.5cm left epididymal cyst (Figure 1). The sonogram also discovered a 9mm hypoechoic right testicular lesion with vascular flow. Given these findings, the patient was counseled on options for management, including radical inguinal orchiectomy, trans-scrotal spermatocelectomy with partial orchiectomy and intraoperative frozen section, or observation with repeat imaging. Given the possibility of testicular cancer, the patient elected for radical orchiectomy with complete removal of the right testicle, epididymis, and spermatic cord via a right inguinal incision. Notable intraoperative findings included a right testicle without obvious evidence of a violation of the tunica albuginea as well as a calcified 2cm para-testicular lesion confined to the epididymis. The patient was discharged immediately post-operatively and had an uneventful recovery. Serum tumor markers were negative. Final pathology revealed a 3.7cm serous cystadenoma of the right epididymis with ovarian-type stroma staining positive for estrogen receptor (ER), progesterone receptor (PR) and Wilms' tumor 1 (WT-1), confirming the ovarian variant. The testis and cord were otherwise unremarkable despite the earlier presence of a right intratesticular lesion on ultrasound. The patient experienced a routine postoperative course. After a review of the benign nature of his pathology, he chose to avoid any office-based active surveillance with labs or imaging and instead would perform routine testicular self-exams at home.

## Discussion

**Figure 2:**
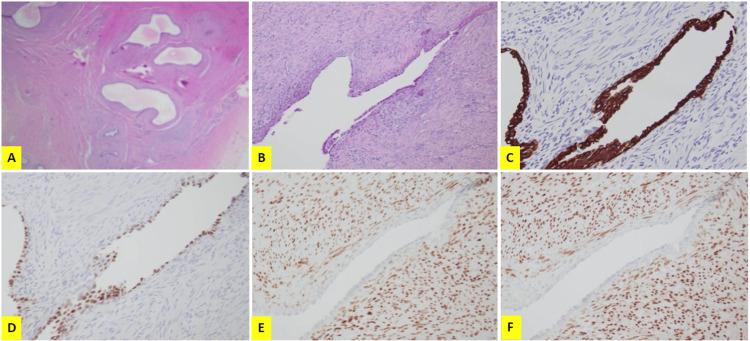
Microscopic appearance of lesion A and B: The lesion arises from the epididymis, consisting of multilocular cystic space lined by a single layer of serous cuboidal or columnar cells with eosinophilic cytoplasm and occasional cilia, no mucinous production or mitotic figures are readily seen (Hematoxylin and eosin; magnification: 20x in A and 200x in B). C and D: Epithelial cells resemble the immunophenotype of gynecologic glands showing diffuse immunopositivity of pankeratin and PAX-8 (C: Cytokeratin AE1/AE3, 200x; D: PAX-8, 200x). E and F:  Ovarian-type stromal cells show diffuse immunopositivity of ER and PR (C: ER, 200x; D: PR, 200x).​

Fewer than 10 identifiable case reports comment on para-testicular serous cystadenomas, which is not to be confused with papillary cystadenoma as it lacks the proper ovarian stains. Our patient represents another case to further display the variable presentation and management strategy of this rare tumor type. The most common presentation is of a testicular mass [1], but other reported sites include the epididymis [2], one bilateral presentation [3] and another isolated to the tunica albuginea [3,4]. In general, this diagnosis is usually made during the third to fifth decade of life, as suspicion for possible testicular malignancy remains high, as does awareness of changes to the genitalia [5]. Nevertheless, patients as young as 12 years old have also been described, adding additional complexity to the clinical decision-making as it pertains to both oncologic control and maintenance of fertility [6]. These masses are rarely described as painful, which likely contributes to their delayed presentation. However, our patient did experience chronic scrotal discomfort prompting his presentation and subsequent diagnosis. The first line management is most often surgical, ranging from excision of the cyst alone to epididymectomy or even radical orchiectomy [7]. These masses historically follow a benign course without any reported elevation of serum tumor markers. On review of the available literature tumor markers are often omitted in the workup likely due to the extra testicular location of these lesions, but even still invasive carcinomas must be excluded. Follow-up is frequently limited with the longest reported follow-up being seven years at which point there was still no evidence of disease recurrence [8]. Diagnosis is based on histology, which describes a simple cyst wall structure composed of columnar ciliated cells expressing vimentin and cytokeratin as demonstrated in Figure 2. They concurrently lack Wolffian Duct CD10 and calretinin expression, which are consistent with fetal Mullerian duct structures [9]. The rarity of this tumor places it low on a differential diagnosis, in favor of more common entities. This includes spermatocele, which classically contains spermatozoa, though they have never been reported to exist within para-testicular serous cystadenomas [8]. The role of biopsy of these lesions is not well established. One center reviewed 63 epididymal cyst biopsies performed over 12 years, a minority of which were consistent with adenomatoid tumor and spermatocele, with no cystadenomas present [6]. Important to note is the gynecologic counterpart to the epididymal serous cystadenoma, which also follows a benign course, suggesting that aggressive post-operative surveillance is likely unnecessary. In summary, primary epididymal serous cystadenomas are a rare entity but can provide a clinical challenge for urologists. The risks and benefits of each management strategy should be discussed to allow the patient to make their best-informed decision. Luckily, this pathology follows a benign course requiring only limited follow-up.

## Conclusions

The differential for masses of the genitalia is broad, especially, within various benign histologic types and this is further limited by an inability of imaging to differentiate solid benign masses from malignant. Our case brings forth yet another unique histologic type of a para-testicular mass, which, in this instance was associated with the epididymis. Given the rarity of these tumors, as well as most of their treatment being based around surgical excision there is not a well-defined management strategy for the undifferentiated patient at the time of original presentation. Fortunately for this population, in our review of a few case reports that do exist, it would seem that regardless of specific location, the ovarian serous cystadenoma sub-type maintains a benign course following surgical excision. Therefore, the need for close postoperative follow-up does not seem to be paramount, however, more cases would need to be established with increased follow-up to confirm this observed trend.
